# The Immune Response of *OAS1*, *IRF9*, and *IFI6* Genes in the Pathogenesis of COVID-19

**DOI:** 10.3390/ijms25094632

**Published:** 2024-04-24

**Authors:** Malena Gajate-Arenas, Ingrid Fricke-Galindo, Omar García-Pérez, Angélica Domínguez-de-Barros, Gloria Pérez-Rubio, Roberto Dorta-Guerra, Ivette Buendía-Roldán, Leslie Chávez-Galán, Jacob Lorenzo-Morales, Ramcés Falfán-Valencia, Elizabeth Córdoba-Lanús

**Affiliations:** 1 Instituto Universitario de Enfermedades Tropicales y Salud Pública de Canarias (IUETSPC), Universidad de La Laguna, 38029 San Cristóbal de La Laguna, Spain; malena.bioresearch@gmail.com (M.G.-A.); omargp6@gmail.com (O.G.-P.); angelica4arealejos@gmail.com (A.D.-d.-B.); rodorta@ull.edu.es (R.D.-G.); 2 HLA Laboratory, Instituto Nacional de Enfermedades Respiratorias Ismael Cosío Villegas, Mexico City 14080, Mexico; ingrid_fg@yahoo.com.mx (I.F.-G.); glofos@yahoo.com.mx (G.P.-R.); rfalfanv@iner.gob.mx (R.F.-V.); 3 Department of Mathematics, Statistics and Operations Research, Faculty of Sciences, Mathematics Section, Universidad de La Laguna, 38200 San Cristóbal de La Laguna, Spain; 4 Translational Research Laboratory on Aging and Pulmonary Fibrosis, Instituto Nacional de Enfermedades Respiratorias Ismael Cosio Villegas, Mexico City 14080, Mexico; ivettebu@yahoo.com.mx; 5 Laboratory of Integrative Immunology, Instituto Nacional de Enfermedades Respiratorias Ismael Cosio Villegas, Mexico City 14080, Mexico; lchavezgalan@gmail.com; 6 Centro de Investigación Biomédica en Red de Enfermedades Infecciosas (CIBERINFEC), Instituto de Salud Carlos III, 28029 Madrid, Spain; 7 Department of Obstetrics and Gynecology, Pediatrics, Preventive Medicine and Public Health, Toxicology, Legal and Forensic Medicine and Parasitology, Faculty of Health Sciences, Universidad de La Laguna, 38200 San Cristóbal de La Laguna, Spain

**Keywords:** antiviral genes, COVID-19, gene expression, immune response, SARS-CoV-2

## Abstract

COVID-19 is characterized by a wide range of clinical manifestations, where aging, underlying diseases, and genetic background are related to worse outcomes. In the present study, the differential expression of seven genes related to immunity, *IRF9*, *CCL5*, *IFI6*, *TGFB1*, *IL1B*, *OAS1*, and *TFRC*, was analyzed in individuals with COVID-19 diagnoses of different disease severities. Two-step RT-qPCR was performed to determine the relative gene expression in whole-blood samples from 160 individuals. The expression of *OAS1* (*p* < 0.05) and *IFI6* (*p* < 0.05) was higher in moderate hospitalized cases than in severe ones. Increased gene expression of *OAS1* (OR = 0.64, CI = 0.52–0.79; *p* = 0.001), *IRF9* (OR = 0.581, CI = 0.43–0.79; *p* = 0.001), and *IFI6* (OR = 0.544, CI = 0.39–0.69; *p* < 0.001) was associated with a lower risk of requiring IMV. Moreover, *TGFB1* (OR = 0.646, CI = 0.50–0.83; *p* = 0.001), *CCL5* (OR = 0.57, CI = 0.39–0.83; *p* = 0.003), *IRF9* (OR = 0.80, CI = 0.653–0.979; *p* = 0.03), and *IFI6* (OR = 0.827, CI = 0.69–0.991; *p* = 0.039) expression was associated with patient survival. In conclusion, the relevance of *OAS1*, *IRF9*, and *IFI6* in controlling the viral infection was confirmed.

## 1. Introduction

Severe Acute Respiratory Syndrome Coronavirus 2 (SARS-CoV-2) is the causative agent of Coronavirus Disease 2019 (COVID-19). In December 2019, the novel SARS-CoV-2 coronavirus was found in hospitalized patients in Wuhan, China. The World Health Organization (WHO) declared COVID-19 as a pandemic on 11 March 2020, and it was declared a global health emergency until May 2023.

SARS-CoV-2 is a large, enveloped, and single-stranded RNA virus. The first targets of SARS-CoV-2 are the epithelial cells from the respiratory tract. As viral replication advances to the lungs, an immune response is promoted, which can trigger critical damage in the lungs if the reaction is excessive and non-coordinated [1,2,3,4].

COVID-19 cases can show a wide variety of symptoms, where asymptomatic and mild symptoms (fatigue, dry cough, fever, headache, anosmia, etc.) are the most common manifestations of the infection [1,5,6]. However, severe cases are characterized by an impaired immune response called “cytokine storm”. This phenomenon is promoted by an increase in proinflammatory cytokines, promoting the influx of immune cells from the circulation to the lungs. Therefore, this overwhelming response results in continuous damage to the lung instead of relieving the infection. Cytokine storm can progress and lead patients to develop acute respiratory distress syndrome (ARDS) and respiratory failure, followed by multi-organ failure, which is the leading cause of mortality in COVID-19 cases [7,8].

The predisposition to severe COVID-19 forms depends on a wide diversity of factors. Aging is one of the most significant, as people over 60 are likely to develop severe forms of the disease [9,10]. Sex is another factor to consider; men have higher ratios of intensive care admission and mortality than women. Furthermore, underlying diseases like obesity, diabetes, cardiorespiratory pathologies, etc., increase the risk of developing ARDS [11,12]. Despite what has been previously mentioned, the phenotypic variability in SARS-CoV-2 infection is not clear enough. Genetic background plays a leading role in the pathogenesis of the virus and host susceptibility. However, studies on the molecular level are necessary to shed light on this issue.

An analysis of the expression of genes related to antiviral activity or inflammatory response can be helpful for the prognosis of a patient infected by SARS-CoV-2. Alterations in gene expression have been linked to a worse disease outcome in respiratory viral infections, including SARS-CoV-2, especially those related to the interferon pathways *IRF9* and *IFI6* [13,14,15]. In a study by Zaas et al., the expression of immune-related genes was determined in individuals infected with rhinovirus, respiratory syncytial virus, and influenza A. Within certain genes, *IFI6* expression in peripheral blood was altered during the infection and enabled the authors to successfully discriminate between infected patients and the healthy control group [16]. Related to SARS-CoV-2 infection, the overexpression of genes related to inflammation has been detected in severe COVID-19 cases [17,18,19]. 

Among the wide range of genes suitable for research, interferon-stimulated genes are particularly interesting. The interferon family is able to induce the synthesis of a wide diversity of proteins involved in antiviral mechanisms [20]. Among these proteins, *OAS1* and *IRF9* have been reported as potential biomarkers for COVID-19 prognosis [21,22]. Moreover, in vitro studies have revealed an inhibitory effect of *IFI6* in viral replication, the current study sheds light on whether this inhibitory effect arises in humans [23,24].

Other kinds of gene that are interesting to study concerning this field of research are chemokines and cytokines. IL-1β is a cytokine found at high levels in ICU patients infected with SARS-CoV-2 [25]. Among chemokines, *CCL5* has been related to cytokine storm in severe COVID-19 cases [26]. Regarding their predominant role in the immune system, *TGFB1* has been linked to pulmonary fibrosis in COVID-19 patients [27].

Based on relevant results reported in the existing literature and a previous study of our group [28], the genes *CCL5*, *OAS1*, *IRF9*, *IFI6*, *TGFB1, IL1B,* and *TFRC* were analyzed in the present study in relation to the severity of COVID-19. 

The information that gene expression can provide is essential to understanding the clinical heterogeneity of the disease and the virus pathogenesis. This study aims to analyze the differential expression of the proposed antiviral and immune response genes among moderate and severe COVID-19 cases to identify a potential biomarker profile that can help improve patient diagnosis, prognosis, and treatment.

## 2. Results

### 2.1. Individuals Included in the Study

Our study group of 160 hospitalized individuals with COVID-19 consisted of 101 men and 59 women, with average ages of 56 and 62 years old, respectively. Comparisons between the main clinical characteristics of the included cases in this study are summarized in Table 1. Within this group, 60.6% of the patients were severe cases. Moderate and severe hospitalized cases had a average ages of 54 and 61 years, respectively (*p* = 0.003), and a higher percentage of individuals over 65 years of age was found among severe patients (*p* = 0.006). Both groups presented a mean of 12 (±7) days from symptom onset until the patient was admitted to the hospital. Moreover, patients with severe disease required more days of hospitalization and invasive mechanical ventilation than cases with moderate disease. A total of 41% of deaths were registered in severe cases in contrast to the 25.9% observed in moderate hospitalized patients. Furthermore, a higher frequency of severe patients (92.7%) needed steroid treatment (*p* < 0.001). No significant differences were found between moderate and severe cases and the rest of the variables.

Moreover, after analyzing the gender effect, non-significant differences were found between the sexes and the clinical variables; it was only observed that men were more likely tobacco smokers (43.6%) than women (11.9%) (*p* < 0.001).

Within the hospitalized patients, 18 individuals presented a chronic respiratory disease. Considering the treatment provided, seven individuals received antibiotics, three required antivirals, six required immunotherapies, and two were treated with convalescent plasma.

### 2.2. Gene Expression Analysis

The gene expression of *TFRC*, *TGFB1*, *IRF9*, *IFI6*, *OAS1*, *IL1B,* and *CCL5* was determined in all the participants. Significant differences in gene expression related to clinical features were observed among hospitalized COVID-19 cases. Patients under 65 years old showed a higher expression of *TFRC* (*p* = 0.002), *CCL5* (*p* < 0.001) and *IFI6* (*p* = 0.006), and those with obesity (BMI ≥ 30) presented higher expression of *OAS1* (*p* = 0.008) and *TGFB1* (*p* = 0.008). Concerning the treatment, lower expression levels of *OAS1* (*p* = 0.014) and *IFI6* (*p* = 0.008) were found in those patients treated with steroids.

**Figure 1:**
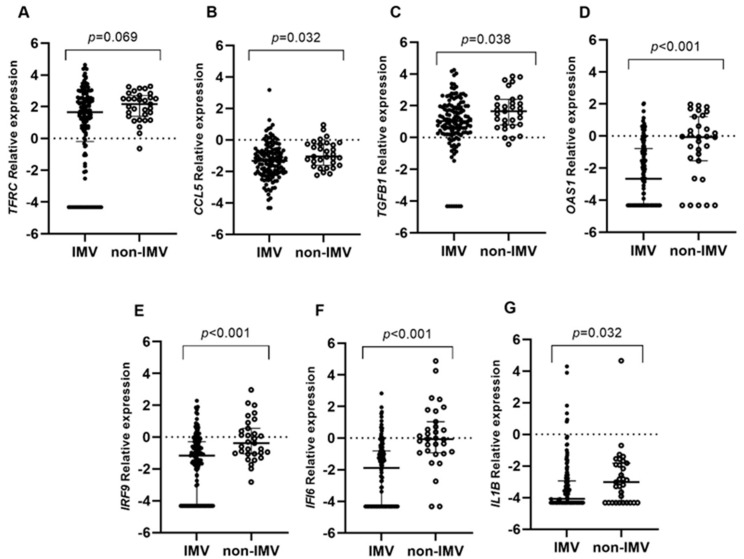
Differential gene expression among invasive mechanical ventilation patients (IMV) and non-invasive mechanical ventilation patients (non-IMV) within the hospitalized group. Lines represent the median with an interquartile range. (**A**) *TFRC*, (**B**) *CCL5*, (**C**) *TGFB1*, (**D**) *OAS1*, (**E**) *IRF9*, (**F**) *IFI6*, and (**G**) *IL1B*. *p*-values < 0.05 were considered significant.

**Figure 2:**
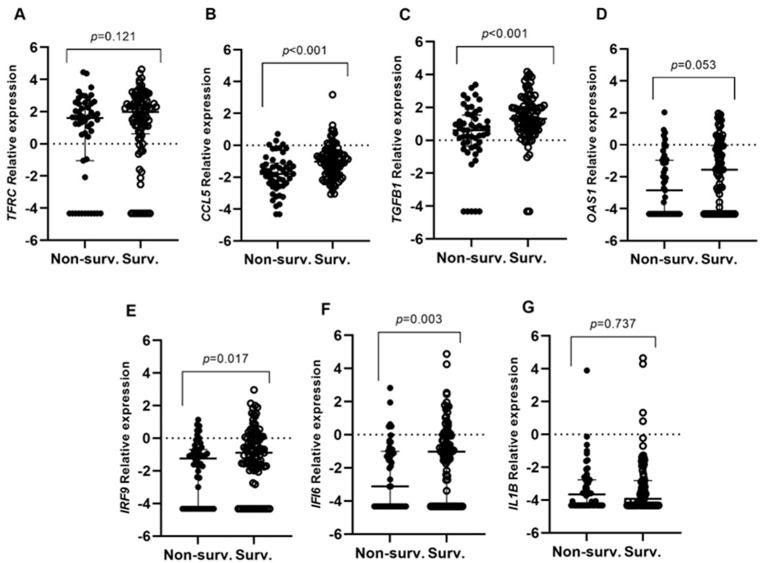
Differential gene expression among patients who underwent hospitalization in relation to survival (Surv vs. Non-surv.). Lines represent the median with an interquartile range (25th–75th pc). (**A**) *TFRC*, (**B**) *CCL5*, (**C**) *TGFB1*, (**D**) *OAS1*, (**E**) *IRF9*, (**F**) *IFI6,* and (**G**) *IL1B*. *p*-values < 0.05 were considered significant.

Interestingly, the expression of *OAS1* (*p* = 0.041) and *IFI6* (*p* = 0.027) was increased in those individuals with moderate disease compared to severe cases. Regarding invasive mechanical ventilation (IMV), increased expression levels were observed for all the studied genes in non-IMV patients, except for *TFRC* (Figure 1A–G). *TFRC*, *OAS1*, *IRF9*, and *IFI6* gene expression was associated with a lower risk of requiring IMV (Table 2). In the same way, the higher expression of *CCL5*, *TGFB1*, *IRF9,* and *IFI6* (Figure 2A–G) observed in survivors hospitalized patients was associated with patient survival (Table 2).

## 3. Discussion

COVID-19 shows a wide range of clinical manifestations where genetic factors can play a relevant role in the development of the disease [29,30]. In the present study, we evaluated the expression of genes related to the immune response and antiviral activity in subjects infected with SARS-CoV-2 with different severity of the disease. Our main findings point towards differences in the expression of the studied genes concerning invasive mechanical ventilation (IMV), and survival in hospitalized cases.

Within the hospitalized patients, moderate cases showed higher expression levels of *OAS1* and *IFI6* than severe cases of the disease, which was also associated with a reduced risk of needing IMV. Moreover, *IFI6* expression was associated with an increase in survival rate. *OAS1* is an interferon-stimulated gene crucial for pathogen control, recognizing viral RNA and promoting RNA degradation via the RNase L pathway [31]. A protective effect against severe forms of COVID-19 has been demonstrated to be associated with this gene [22,32,33]. *IFI6* is another interferon-stimulated gene and a mitochondrial-target protein that regulates apoptosis [34]. SARS-CoV-2 infection promotes *IFI6* expression [15], but an inhibitory effect in hepatitis C virus infection and Ebola virus replication has also been suggested according to the expression of this gene [23,24]. This fact might explain the relationship between *IFI6* expression and the lower incidence of severe outcomes. However, it is essential to remark that both genes present polymorphisms that can alter their expression, and they have not been considered in this study [22,35].

*IRF9* is an interferon-stimulated gene; its deficiency has been related to worse outcomes in respiratory viral infections [36,37]. In human airway cultures, *IRF9* expression is upregulated in response to SARS-CoV-2 infection [38]. Moreover, a previous study from us reported higher expression of *IRF9* in the upper airways of mild cases of SARS-CoV-2 [28]. This current research found an association of *IRF9* expression with a lower risk of IMV and survival increase. Regarding this finding, we can confirm that *IRF9* plays a central role in SARS-CoV-2 infection. *IRF9* deficiency was associated with impaired control of other viral diseases and may act as a risk biomarker of COVID-19 [21,36]. Furthermore, it is well known that SARS-CoV-2 can alter the expression of certain genes for its own benefit. For example, one of the host immune evasion strategies is the suppression of interferon pathways that are effective against viral infections [39,40].

TGF-β1 is the predominant isoform expressed in the immune system and is involved in cell proliferation, differentiation, migration, and survival [41]. According to the literature, higher levels of TGF-β1 detected in lung tissue were associated with pulmonary fibrosis in COVID-19 patients [27,42]. Our study showed an association between increased *TGFB1* expression and lower mortality risk. Higher levels of *TGFB1* were found in hospitalized patients who survived this infective disease compared to non-survivors. In the same way, in a study performed by Kang et al., decreased TGF-β1 concentrations were found in COVID-19 patients with fatal outcomes [43]. Moreover, transgenic mice knocked down for *TGFB1* had premature death due to an excessive inflammatory response. Our results confirm what was already reported for TGF-β1; it can act as an immunosuppressor of proinflammatory cytokines, preventing an uncontrollable inflammatory response [44].

CCL5, also called RANTES (Regulated upon Activation, Normal T Cell Expressed and Presumably Secreted), is a chemokine that belongs to the C-C chemokine subfamily. Being produced by several cell types (platelets, macrophages, fibroblasts, etc.) and promoting the migration and recruitment of immune cells, it has been related to multiple biological processes like pathogen control, cancer, and atherosclerosis [45]. Studies performed on *CCL5* with COVID-19 should be considered with caution. The upregulation of *CCL5* has been related to cytokine storm, and high levels of CCL5 in plasma have been found in critical COVID-19 patients [26,46]. However, a low expression of *CCL5* in upper airway studies has been associated with worse outcomes [47,48]. Our study revealed that surviving patients and those who did not require IMV expressed higher levels of *CCL5*. Moreover, we found an association between the expression of *CCL5* and a lower risk of death. These data suggest once more that the early expression of *CCL5* plays an essential role in controlling viral replication and macrophage survival, preventing a prolonged inflammatory response, and controlling viral infection [45].

In SARS-CoV-2 infection, the massive synthesis of cytokines leads to cytokine storm, in which an excessive pro-inflammatory response can lead to lung injury instead of removing the infection [8,49]. One of the most critical pro-inflammatory cytokines of the innate immune response is 1L-1β [8]. Higher levels of IL-1β in plasma have been reported in plasma from hospitalized patients and subjects with post-acute sequelae [25,50]. However, our study did not find significant differences among groups, but increased expression levels of *IL1B* were observed in non-IMV patients. SARS-CoV-2 infection has been associated with lower levels of IL-1β when compared to other respiratory viruses, probably because inflammasome pathways are non-responsive in SARS-CoV-2 infection, decreasing the synthesis of IL-1β [15].

*TFRC* is a gene that encodes the TfR1 protein, a cell surface receptor that transports iron from the outside to the inside of the cell via receptor-mediated endocytosis. Alteration in the levels of *TFRC* has been associated with malignant forms of certain cancers [51]. Concerning COVID-19, Muhammad et al. (2022) did not observe significant differences in *TFRC* expression between asymptomatic–mild cases and the severe group [52]. However, we found a higher expression of *TFRC* associated with a lower risk of IMV. In agreement with this finding, previous research by our group reported a higher gene expression of *TFRC* in the upper airways of mild COVID-19 cases [28]. An in silico analysis showed that *TFRC* could be affected by SARS-CoV-2 infection through the *ACE2* interaction network [53].

Clinical features affect the expression of genes. One of the most relevant is age. It is known that *ACE2* expression and the apoptotic process are influenced by age [10]. In the current study, we observed a higher expression of *CCL5* and *IFI6* in individuals under 65. Both genes have been associated with a lower risk of death, promoting a protective effect that in elderly patients could be altered. *TFRC* expression has been shown to be higher in this range of age. *TFRC* has an indirect relationship with *ACE2* that influences its expression, but at the same time, *ACE2* is affected by aging [10]. Obesity is considered one of the major risk factors for COVID-19. Multiple studies show an increase in gene expression related to the immune system. Our study shows a higher expression of *OAS1* and *TGFB1* in individuals with obesity. Obesity can impact the innate and adaptative immune responses, promoting pro-inflammatory pathways and triggering severe forms of COVID-19 [54]. On the other hand, patients treated with steroids showed lower expression levels of *OAS1* and *IFI6*. Steroids can modulate the immune response, avoiding excessive inflammation; alterations have been reported in the expression of interferon-stimulated genes in patients undergoing steroid treatment [55,56].

Our study is not exempt from limitations. Firstly, the sample collection period was ample, potentially including different SARS-CoV-2 variants we did not consider. Second, the analysis of the different fractions of blood cells was beyond the scope of this research, but we are aware that some genes express differently within different leucocyte cells. Third, gene expression may differ between different types of tissues, which may be the case for gene expression in inflammatory cells and lymphoid organs during the immune response in contrast to peripheral blood. Last, replication on an independent cohort is needed to clarify our results, especially the contradictory results.

In conclusion, the expression of *IFI6*, *OAS1*, and *IRF9* was shown to be associated with a lower risk of death and IMV. The expression of these genes could show a protective effect against SARS-CoV-2 infection. Paradoxically, we found that the expression of *TGFB1* and *CCL5* is associated with the survival of patients. Future prospective studies in a larger cohort are necessary to confirm the present findings.

## 4. Materials and Methods

### 4.1. Patients and Study Samples

A total of 160 individuals with a COVID-19 diagnosis hospitalized in the Instituto Nacional de Enfermedades Respiratorias Ismael Cosio Villegas (INER) in Mexico City, Mexico, were included in this study. Subjects with a hospital admission PaO_2_/FiO_2_ ratio above 100 were considered moderate cases, while subjects with a PaO_2_/FiO_2_ ratio under 100 were considered severe cases [57]. The body mass index (BMI) was categorized using the parameters from the Centres for Disease Control and Prevention (https://www.cdc.gov/healthyweight/assessing/bmi/adult_bmi/index.html accessed on 20 May 2023). Only 18% of the patients had receive vaccinations, with 5% of them having received a second boost.

Whole-blood samples were collected from all the subjects infected with SARS-CoV-2 between July 2020 and January 2023. The mean number of days from symptom onset to sample collection was 27 ± 11. The diagnosis of COVID-19 was carried out by reverse transcription–polymerase chain reaction (RT-qPCR) from upper airway samples. 

All the clinical data were registered, and informed consent was obtained from every participant in the study. The study was approved by the Institutional Ethical Research and Investigation Committees from INER (C53-20). All the procedures were performed following the Declaration of Helsinki.

### 4.2. Gene Expression Study

The following genes related to the host immune response and inflammatory process were analyzed: *CCL5*, *OAS1*, *IRF9*, *IFI6*, *TGFB1*, and *IL1B*. *TFRC* was also selected due to our previous findings (Gajate-Arenas et al., 2023 [28]). 

RNA isolation was carried out using Qiazol Lysis Reagent (Qiagen^TM^, Hilden, Germany) and TRIzol™ Reagent (Invitrogen^TM^, Carlsbad, CA, USA), and its quality was evaluated by NanoDrop Lite (Thermo Fisher Scientific, Waltham, MA, USA). The relative gene expression analysis was set up in two-step RT-qPCR. First, RNA was retrotranscribed into cDNA using the SuperScript^TM^ VILO^TM^ cDNA Synthesis Kit (Invitrogen^TM^, Carlsbad, CA, USA) following the manufacturer’s instructions. Second, qPCR was performed using the TaqMan™ Gene Expression Master Mix and TaqMan™ Gene Expression Assays (Thermo Fisher Scientific, Applied Biosystem, Waltham, MA, USA). The reaction was performed in a real-time qPCR machine QuantStudio 5 (Thermo Fisher Scientific, Applied Biosystem, Waltham, MA, USA). Each reaction was performed in duplicate, setting up the experiment in 40 cycles. For data normalization, the *ACTB* housekeeping gene was used (resulting the most stable gene analyzed with NormFinder and Bestkeeper v.1 software, https://www.moma.dk/software/normfinder, accessed on 15 May 2021, and https://www.gene-quantification.de/bestkeeper.html, accessed on 15 May 2021). Relative expression analysis of the target genes was performed using the comparative threshold method 2^ΔΔ^Ct.

### 4.3. Statistical Analysis

Continuous variables were described using means and standard deviation when normally distributed and medians and percentiles (P_25_; P_75_) when not normally distributed, and categorical variables using frequency and percentage. Outliers in the data were assessed through the inspection of boxplots, and, when necessary, the normality distribution of the variables was evaluated by a Shapiro–Wilk test or Kolmogorov–Smirnov test as appropriate (*p* > 0.05). Data were normalized using the method of log two-fold and absolute gene-wise changes in expression. Parametric tests (*t*-test and ANOVA) and non-parametric tests (the Mann–Whitney U test and the Kruskal–Wallis test) were used for group comparisons as appropriate. A *t*-test was carried out for age, and ANOVA was performed to measure the effect of age among groups. A Chi-squared test was performed to determine differences in gene expression and clinical variables among groups.

To determine predictor variables for survival and IMV, a binary logistic regression model, one for each, was fitted. Age, sex, and normalized gene expression were considered as predictor variables. All assumptions for binary logistic regression were checked. Using the forward Likelihood Ratio method, the final models retained all predictor variables significantly associated with the outcomes (*p* < 0.05). Odds ratios (OR) with 95% confidence intervals were reported. A Hosmer and Lemeshow goodness-of-fit test (*p* > 0.05) was used to check model fitness.

Statistical analyses were performed using SPSS v. 25 (IBM Corp, New York, NY, USA) and GraphPad Prism v. 9.4.1 (Dotmatics, Boston, MA, USA) software.

## Figures and Tables

**Table 1:** Demographic and clinical characteristics of hospitalized individuals with COVID-19.

Characteristics	Moderate (*n* = 63)	Severe (*n* = 97)	*p*-Value
Age, years (mean ± SD)	53.9 ± 13.5	60.9 ± 15.1	0.003
Age category			0.003
● <65 years	50 (79.4%)	55 (56.7%)	
● 65 years	13 (20.6%)	42 (43.3%)	
Sex			0.278
● Men (%)	43 (68.3%)	58 (59.8%)	
● Women (%)	20 (31.7%)	39 (40.2%)	
Tobacco smoking (%)	23 (36.5%)	28 (28.9%)	0.311
BMI (median (P_25_–P_75_))	27.9 (25.5–32.4)	28.5 (25.3–33.4)	0.407
BMI category			0.647
● Normal (21–24) (%)	13 (21.0%)	20 (21.3%)	
● Overweight (25–29) (%)	23 (37.1%)	33 (35.1%)	
● Obese (≥30) (%)	25 (40.3%)	41 (43.6%)	
Hospitalization days (median (P_25_–P_75_))	16.5 (11–35.5)	36 (23.7–58)	<0.001
PaO_2_/FiO_2_ (mean ± SD)	289.9 ± 55.5	72.1 ± 17.2	<0.001
IMV (%)	32 (50.8%)	97 (100%)	<0.001
IMV days (median (P_25_–P_75_))	1.5 (0–21.7)	28.5 (17.7–41)	<0.001
Outcome			0.055
● Survival (%)	43 (74.1%)	51 (58.6%)	
● Non-survival (%)	15 (25.9%)	36 (41.4%)	
Type 2 diabetes (%)	20 (31.7%)	33 (34.0%)	0.765
Hypertension (%)	20 (31.7%)	39 (40.2%)	0.278
Chronic respiratory disease (%)	10 (15.9%)	7 (7.2%)	0.083
Steroid treatment (%)	44 (69.8%)	89 (92.7%)	<0.001

Data are expressed as n (%), mean ± SD, or median (P_25_–P_75_). BMI, body mass index; IMV, invasive mechanical ventilation. *p*-values < 0.05 were considered significant.

**Table 2:** Binary logistic regression analysis showing adjusted effect of differential expression of studied genes.

Gene	OR	CI (95%)	*p*-Value
**Survival**			
*TFRC*	-	-	ns
*CCL5*	0.574	0.396–0.832	0.003
*TGFB1*	0.646	0.500–0.835	0.001
*OAS1*	-	-	ns
*IRF9*	0.800	0.653–0.979	0.030
*IFI6*	0.827	0.690–0.991	0.039
*IL1B*	-	-	ns
**IMV**			
*TFRC*	0.787	0.620–0.999	0.049
*CCL5*	-	-	ns
*TGFB1*	-	-	ns
*OAS1*	0.642	0.516–0.798	0.001
*IRF9*	0.581	0.427–0.790	0.001
*IFI6*	0.544	0.391–0.688	<0.001
*IL1B*	-	-	ns

OR = odds ratio; CI (95%) confidence interval at 95%. *p*-values < 0.05 were considered significant. ns = non-significant. In the binary logistic regression model, age, sex, and normalized gene expression were considered predictor variables.

## Data Availability

Data are available upon reasonable request. All data relevant to this study are included in the article or have been uploaded as additional information.
